# The Role of Medical Mistrust in Concerns about Tumor Genomic Profiling among Black and African American Cancer Patients

**DOI:** 10.3390/ijerph19052598

**Published:** 2022-02-23

**Authors:** Ariel Hoadley, Sarah Bauerle Bass, Yana Chertock, Jesse Brajuha, Paul D’Avanzo, Patrick J. Kelly, Michael J. Hall

**Affiliations:** 1 Risk Communication Laboratory, Department of Social and Behavioral Sciences, College of Public Health, Temple University, 1301 Cecil B Moore Ave, Rm 947, Philadelphia, PA 19122, USA; sbass@temple.edu (S.B.B.); jesse.aaron.bee@gmail.com (J.B.); paul.davanzo@temple.edu (P.D.); patrick.kelly@temple.edu (P.J.K.); 2 Fox Chase Cancer Center, Cancer Prevention and Control Program, Department of Clinical Genetics, 333 Cottman Avenue, Philadelphia, PA 19111, USA; yana.chertock@fccc.edu (Y.C.); michael.hall@fccc.edu (M.J.H.)

**Keywords:** tumor genomic profiling, precision medicine, medical mistrust, cancer health disparities, cluster analysis

## Abstract

Tumor genomic profiling (TGP) is used in oncology practice to optimize cancer treatment and improve survival rates. However, TGP is underutilized among Black and African American (AA) patients, creating potential disparities in cancer treatment outcomes. Cost, accuracy, and privacy are barriers to genetic testing, but medical mistrust (MM) may also influence how Black and AA cancer patients perceive TGP. From December 2019 to February 2020, 112 Black and AA adults from two outpatient oncology sites in Philadelphia, PA without a known history of having TGP testing conducted completed a cross-sectional survey. Items queried included sociodemographic characteristics, clinical factors, patient–oncologist relationship quality, medical mistrust, and concerns about TGP. A k-means cluster analysis revealed two distinct psychographic clusters: high (MM-H) versus low (MM-L) medical mistrust. Clusters were not associated with any sociodemographic or clinical factors, except for age (MM-H patients older than MM-L patients, *p* = 0.006). Eleven TGP concerns were assessed; MM-H patients expressed greater concerns than MM-L patients, including distrust of the government, insurance carriers, and pharmaceutical companies. TGP concerns varied significantly based on level of medical mistrust, irrespective of sociodemographic characteristics. Targeted communications addressing TGP concerns may mitigate disparities in TGP uptake among those with medical mistrust.

## 1. Introduction

Traditional germline genetic testing sequences non-cancerous cells to evaluate whether an individual has inherited mutations associated with increased risk of developing disease, including cancer [1]. In contrast, tumor genomic profiling (TGP), also known as tumor molecular profiling, uses next-generation sequencing to test tumor samples and/or other cancerous cells to identify acquired mutations that can serve as therapeutic targets and aid in the treatment decision-making process [2,3,4,5] with the goal of increasing survival [4,6], improving treatment response [7], maintaining quality of life, and limiting exposure to ineffective therapies with adverse side effects [8,9,10]. TGP results can also provide cancer patients with additional options, such as determining eligibility for participating in clinical trials [11,12].

As a form of precision medicine, TGP is increasingly used in routine oncology practices in the United States [8,11,13]. For example, using Surveillance, Epidemiology, and End Results (SEER) registry data, Zhang and colleagues (2020) found that Oncotype DX (ODX), an early form of TGP used to guide breast cancer treatment decisions, had increased significantly in use from 2004 to 2015 among US adults with lymph node-negative breast cancer as well as adults with one to three positive lymph nodes (2.0% to 42.7%; 0.3% to 27.9%, respectively) [14]. In recent years, the complexity of TGP testing has grown markedly, with many tests now reporting results across hundreds of tumor and germline genes and incorporating DNA, RNA, and other data into their diagnostic reports. Increasing use of TGP testing in cancer treatment is tied to both the therapeutic value of TGP and the introduction of the Precision Medicine Initiative (PMI), developed by the US National Institutes of Health (NIH) and the National Cancer Institute. PMI launched in 2015 with one objective being to use genomics to improve the quality of oncology prevention and treatment [15,16].

Given that TGP is being increasingly implemented within oncology practices, understanding patients’ attitudes and concerns about TGP is a priority. However, much of the extant research on TGP has limited generalizability due to underrepresentation of participants from racial and ethnic minority groups and lower socioeconomic status, and/or the inability to perform meaningful analyses in diverse subgroups due to small sample sizes [17,18,19]. The significant increase in use of TGP has been predominantly seen among non-Hispanic, white cancer patients with high educational attainment and household income [20,21,22]. Moreover, despite the growing importance of TGP across cancer disease sites, Black and African American (AA) cancer patients undergo TGP at significantly lower rates, even after controlling for other sociodemographic and clinical factors [20,23]. In addition, a 2018 systematic review found that none of the eleven studies that investigated the role of tumor genetic profiles associated with survival by race considered the potential significance of social, environmental, or systemic factors as moderating factors [24].

In deciding whether to proceed with TGP, the potential benefits of testing are weighed against the potential harms, costs, and/or undesired consequences of having TGP and receiving its results. For example, while TGP may provide the oncologists with additional information on treatment options, many findings may not be clinically actionable or may not lead to a change in treatment, resulting in unrealized expectations and diminished hope [25,26]. Other barriers related to TGP include fears that findings can result in incidental secondary germline hereditary findings [19], such as finding mutations associated with a heritable form of early onset dementia [27] or other types of cancer [28]. Other barriers to TGP exist, including limited knowledge, awareness, outcome expectations, and minimal perception of cancer risk [29,30]. Interpreting TGP requires at least modest knowledge of genetics to make informed decisions about TGP, and this knowledge influences the ability to communicate wishes to a provider. However, health, cancer, and genetic literacy impact how TGP is perceived and understood. Lower levels of these three types of literacy are also associated with less health activation and health disparities [31] and have been shown to be more prevalent in ethnic and minority patients [32,33,34]. In addition, researchers postulate that medical mistrust hinders the development of health literacy among Black and AA individuals [35]. This creates a consequential divide between those who may or may not benefit from TGP. For example, when patients have higher levels of cancer health literacy, they also report greater self-efficacy in their cancer treatment decision-making processes [32] and greater health-related quality of life [36]. Moreover, higher levels of cancer health literacy are inversely related to number of inpatient hospitalizations and length of hospitalization stays [37]. However, given the disproportionately lower rates of TGP use among Black and AA cancer patients and the lower levels of genetic literacy, understanding the specific barriers patients face during their cancer treatment should be addressed to close the health disparities gap [38]. 

Medical mistrust is an important potential barrier to use of TGP in Black and AA cancer patients and stems from the confluence of historical and ongoing systemic injustices, inequities, and systemic disadvantages imposed disproportionately on these communities; this limits the trustworthiness of participating organizations and systems, including the US healthcare system, healthcare organizations, insurance companies, pharmaceutical companies, and the government [39]. Consequentially, medical mistrust and concerns about privacy, stigmatization, and discrimination based on genetic test results are commonly found [30]. Other studies have also found that past negative healthcare experiences and expectations of experiencing racism in healthcare settings were strong predictors of greater medical mistrust and healthcare avoidance [40,41].

Historically, medical mistrust has been used to explain why Black and AA patients participate less frequently in clinical trials than white patients [42,43]. Discussions about medical mistrust have more recently changed, shifting the imperative away from changing individual-level mistrust to transforming healthcare systems to be more trustworthy [44] as well as other institutional and/or systemic changes [39]. Preliminary research has investigated the relationship between medical mistrust and attitudes and beliefs about TGP among Black and AA adults, although study samples have been limited to specific subgroups, such as men [45] and advanced-stage cancer patients [46]. No research has looked at a broader group of Black and AA cancer patients to assess whether medical mistrust alone is related to concerns about TGP, irrespective of sociodemographic or clinical characteristics.

Thus, the objective of this study was to understand the role of medical mistrust in perceptions of TGP in Black and AA cancer patients by using psychographic segmentation to understand perceptions of TGP by level of medical mistrust. The aim of the research is to create better communication tools clinicians can use to address the unique barriers to TGP in Black and AA cancer patients.

## 2. Materials and Methods

### 2.1. Study Recruitment and Ethics

Participants were recruited from December 2019 to February 2020 from two academic oncology practices in Philadelphia, PA associated with Temple Health/Fox Chase Cancer Center. One is a suburban cancer center and the other an urban cancer center associated with a large academic safety net hospital and comprehensive cancer center. Prospective participants were recruited either in-person from waiting rooms and treatment suites or invited via letter to complete an electronic survey. Patients had the choice to complete the survey on an electronic tablet or on a paper survey, including informed consent. Participants were compensated USD 15 in exchange for their participation. All study procedures were approved by Fox Chase Cancer Center’s Institutional Review Board (IRB18-8006).

### 2.2. Study Population and Demographics

**Figure 1:**
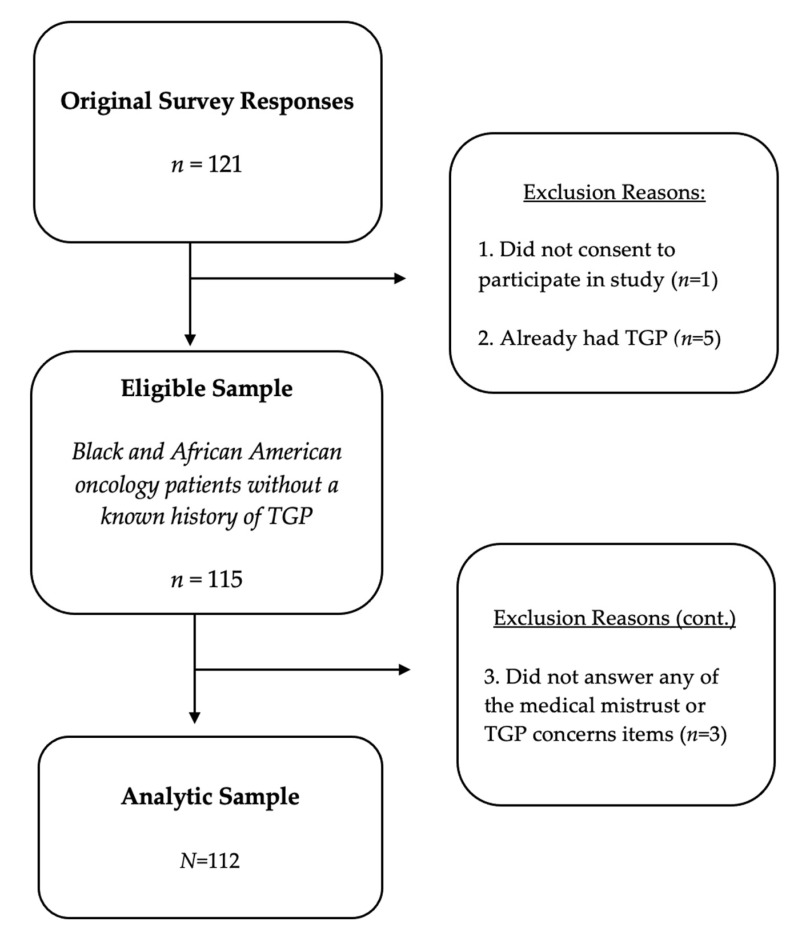
Study inclusion flow chart for analytical sample (*N* = 112).

There were originally 121 surveys collected, from which the analytic sample was limited to *N* = 112 self-reported Black and/or AA adult oncology patients from the Philadelphia metropolitan area who provided informed consent to participate in the study and who self-reported no history of TGP or being unsure whether they had ever had TGP. To assess history of TGP, a single item was asked: “Have you ever had tumor genetic testing? (Tumor genomic profiling (TGP) testing is a test that doctors perform on a patient’s tumor to find a new treatment, usually for advanced cancer).” The three response options were: (1) yes, (2) no, and (3) unsure. The analytical sample included 79.5% (*n* = 89) who had never had TGP and 20.5% (*n* = 23) who were unsure if they had ever had TGP. Figure 1 displays the study inclusion flow chart for the present study’s analytical sample.

### 2.3. Study Materials and Survey Development

To increase the validity of this study and related results, study materials, including survey items, were reviewed by a patient advisory board, all of whom were Black and/or AA cancer patients, prior to survey administration.

### 2.4. Medical Mistrust

Combining insights from focus groups [47] and items from the Group-Based Medical Mistrust Scale [48], ten items were developed to assess mistrust of the healthcare system. Most items measured racism-based medical and institutional mistrust, but some items assessed medical mistrust more broadly. Other items assessed mistrust of science, researchers, pharmaceutical companies, government and regulatory bodies, and insurance companies. Response options were on an 11-point Likert-type scale, with values ranging from a low rating of “1” reflecting strong disagreement to a high rating of “11” reflecting strong agreement. The wording of all mistrust items is displayed in Table 1.

### 2.5. Sociodemographic Characteristics

Sociodemographic characteristics included participant gender which was assessed by asking, “What is your gender?” Participants were provided with six response options, including man, woman, transgender man, transgender woman, gender non-binary/non-conforming, or another gender. However, no participants reported a gender identity other than man or woman, so the gender variable used in analyses was a dichotomous variable. Educational attainment was trichotomized as: (1) high school/GED or less, (2) vocational school or some college, and (3) college degree or more.

Participants were also asked about their annual household income across all sources. Responses were grouped into four categories: (1) less than USD 10,000, (2) USD 10,000 to USD 25,000, (3) USD 25,001 to USD 75,000, and (4) more than USD 75,000. Finally, participants selected all that applied from a list of healthcare insurance options. While participants were given a response option for not having healthcare insurance, all participants reported having at least one form of healthcare insurance. In analyses, insurance status was categorized into whether the respondent had (1) Medicaid coverage only, (2) Medicare or dual eligibility (Medicare and Medicaid), or (3) all other insurance coverage types.

### 2.6. Clinical Factors

Clinical factors included participants’ oncology treatment facility, cancer history, and current cancer stage. Participants were asked if they were current patients at either site location, or neither treatment facility. A single item was used to assess cancer history, specifically about any prior diagnosis of cancer. Response options were dichotomous (i.e., yes/no). To assess participants’ current stage of cancer, participants were asked, “What stage of cancer do you currently have?” Response options included Stage 1, Stage 2, Stage 3, Stage 4, cancer survivor, or unsure. For statistical analyses, responses were grouped into four categories: Stages 1 through 3, Stage 4 (Advanced/Late Stage), Cancer survivor, and Unsure.

### 2.7. Relational Factors

Three items were included to evaluate the quality of relationships between patients and their oncologists. Items were developed based on findings from five focus groups with Black and AA patients from the study sites [47] and were further informed by constructs from the Consultation and Relational Empathy Measure (CARE) [49]. To assess patients’ level of comfort talking with their oncologist, patients were given the statement, “I feel comfortable talking with my oncologist (main cancer doctor).” Other items included, “I feel my oncologist listens to me and does not rush me” and “My oncologist listens to patients equally, regardless of race.” All relational items’ responses were on an 11-point Likert-type scale and ranged from a rating of “1” reflecting strong disagreement to a rating of “11” reflecting strong agreement. Relational items are displayed in Table 2.

### 2.8. Concerns about Tumor Genomic Profiling

Eleven items were developed based on qualitative insights generated from five focus groups with Black and AA patients from the study sites [47]. Items assessed patients’ specific concerns about tumor genomic profiling. For example, participants were provided with the statement, “I am concerned about what my tumor genetic test might show.” Other items reflected concerns about data privacy and confidentiality, insurance discrimination, clinical utility of test results, quality of provider communication about test results, accuracy of test results, implications of test results, and other discomfort. Response options were on the same 11-point Likert-type scale noted above. TGP concern items are displayed in Table 3.

### 2.9. Statistical Analysis

A k-means cluster analysis was conducted to identify psychographic clusters among the sample of Black and AA oncology patients without a known history of TGP. K-means clustering is an unsupervised, Euclidean distance-based approach to segmentation analysis that classifies cases into groups with shared features [50]. A two-cluster solution was specified, missing responses were excluded pairwise, and classification was based on ten medical mistrust items.

In bivariate analyses, chi-squared tests of independence were used to examine nominal variables by medical mistrust cluster. Chi-square tests of independence were also used to evaluate differences in patients’ sociodemographic characteristics between the suburban and urban clinical sites among a subgroup of patients who reported being a patient at only one of the clinical sites. Fisher’s exact test was used if an expected cell count was less than five, which violated the assumptions of a chi-squared test. Independent samples t-tests were used to evaluate mean differences in patients’ age, the three patient–provider relationship items, and all eleven items for concerns about TGP by medical mistrust cluster. Missing responses were excluded pairwise.

## 3. Results

Sociodemographic characteristics differed between the suburban and urban clinical sites. For example, 53.2% of patients from the suburban clinical site had a college degree or higher educational attainment compared to 14.3% of patients from the urban site. In addition, 62.9% of patients from the urban site had a high school degree or less as compared with 17.7% of patients from the suburban site, χ^2^(2, *N* = 97) = 22.361, *p* < 0.001. Similarly, 34.5% of patients from the suburban site reported an annual household income of more than USD 75,000 as compared with only 3.4% of urban clinical site patients, χ^2^(3, *N* = 84) = 22.374, *p* < 0.001. A significantly greater percentage of patients from the urban clinical site had Medicare and/or Medicaid health insurance coverage compared to patients from the suburban site (62.9% vs. 35.3%, χ^2^(1, *N* = 97) = 6.763, *p* = 0.009). Finally, patients from the urban clinical site were older, on average, than suburban clinical site patients (62.1 years vs. 56.7 years), *F*(1,94) = 5.90, *p* = 0.017.

### 3.1. Medical Mistrust Clusters

The k-means cluster analysis yielded two clusters. The first cluster included 39.3% (*n* = 44) of the sample and was comprised of patients with low medical mistrust (MM-L). The second cluster had 60.7% (*n* = 68) of the sample and was comprised of patients with high medical mistrust (MM-H). Mean levels of all ten medical mistrust items by medical mistrust cluster are shown in Table 1.

### 3.2. Sociodemographic, Clinical, and Relational Factors by Medical Mistrust Cluster

The MM-L and MM-H clusters were similar across sociodemographic variables (Table 2). While the percentage of the MM-H cluster with a college degree was 17 points more than the MM-L cluster (42.6% vs. 25.0%, respectively), no significant associations were found between medical mistrust clusters and gender (*p* = 0.50), educational attainment (*p* = 0.14), annual household income (*p* = 0.80), or Medicaid and/or Medicare status (*p* = 0.99). However, patients with high medical mistrust (MM-H) were older, on average, than those with low medical mistrust (60.9 years vs. 55.4 years, respectively; *p* = 0.006).

With respect to clinical factors, there were no significant differences in participants’ history of cancer (*p* = 0.28), cancer stage (*p* = 0.34), or oncology treatment facility (*p* = 0.79) between medical mistrust clusters. Finally, there were no significant differences between medical mistrust clusters on two of the three items assessing relational factors (*p* > 0.05). While ratings were still very high between both clusters, the MM-L group felt slightly more comfortable with their oncologist than the MM-H cluster (10.8 vs. 10.4, *p* = 0.026).

### 3.3. TGP Concerns by Medical Mistrust Cluster

TGP concerns stratified by MM cluster can be seen in Table 3. Compared to patients in the low medical mistrust (MM-L) cluster, patients in the high medical mistrust (MM-H) cluster reported greater concerns about TGP, including testing cost (*p* = 0.002), insurance discrimination (*p* < 0.001), privacy breaches of test results to external entities (*p* = 0.002), clinical utility of test results (*p* < 0.001), accuracy of test results (*p* = 0.001), privacy breaches of test results for use by researchers (*p* < 0.001), physical discomfort of testing (*p* = 0.005), and provider communication skills about inconclusive test results (*p* = 0.029). There were no significant differences between groups for concerns about what TGP test results might show (*p* = 0.249) or incidental germline findings (*p* = 0.077).

## 4. Discussion

In the present study, higher levels of medical mistrust were associated with greater concerns about tumor molecular profiling among Black and AA oncology patients. This is consistent with earlier findings that higher levels of medical and institutional mistrust are associated with underutilization of genetic counseling and other cancer care services among Black women with hereditary risk for developing breast and ovarian cancer [51].

TGP concerns varied considerably by medical mistrust. Differences in concerns about TGP cost and health insurance discrimination were seen. Notably, there were no significant differences in socioeconomic indicators (i.e., educational attainment, type of health insurance coverage, and annual household income) between medical mistrust clusters (*p* > 0.05) or between clusters and study sites. This consistent association of MM-H to concerns related to cost and insurance was seen despite the significantly different sociodemographic characteristics between the two clinical sites, one of which serves a federally designated persistent poverty area in North Philadelphia and whose population is mainly Medicaid/Medicare insured, and the other which has a patient population that is more likely to be privately insured and have a higher socioeconomic status. This finding underlines the importance of medical mistrust as a psychographic construct in Black and AA patients: MM-H was associated with having greater concerns about TGP cost and its potential for creating health insurance discrimination, despite both the MM-H and MM-L groups being similarly insured.

The relevance of cost concerns to medical mistrust could be rooted in experiences with non-transparent medical payment processes and/or out-of-network “surprise billing” [52], meaning that a patient receives a large and unexpected medical bill from an out-of-network provider, diagnostic or lab facility, or other healthcare entity [53]. The association of mistrust with cost concern could also stem from perceptions of higher costs associated with advanced diagnostic testing such as germline genetic and tumor genomics [54]. These unexpected medical costs place significant financial and psychological burdens upon patients and result in lingering concerns about future healthcare use and insurance billing practices [55], concerns which may be further magnified in patients with lower health literacy [56]. The present study also had only a small percentage (13.4%) of patients who had solely Medicaid coverage. While the Centers for Medicare & Medicaid issued a national coverage determination for next-generation sequencing in 2018 [57], underutilization of TGP persists among Medicaid beneficiaries whose coverage remains limited and is determined at the local/state level [58,59].

Higher medical mistrust was also associated with concern for privacy breaches, both to external entities and more specifically to medical researchers. With multiple examples now in the public domain of racist and harmful medical practices by private and federal institutions toward AA and Black Americans [60,61], concerns among AA and Black Americans about trusting these same institutions to safeguard the privacy of genetic testing data are certainly not surprising. This study also did not find significant differences in level of medical mistrust by sociodemographic factors, which differed slightly from previous research that has identified socioeconomic factors as significantly associated with level of medical mistrust [62,63]. There may be alternate explanations for why there were no significant differences on socioeconomic indicators between medical mistrust clusters. First, participants examined in the current study reported active health insurance and had received medical care at one or more oncology centers, suggesting that access and engagement with health services was already high among this sample, which may not generalize to other Black and AA oncology patients with fewer resources or with greater levels of medical mistrust or healthcare avoidance. It is also plausible that medical mistrust develops and exists independently of socioeconomic indicators, which is consistent with other research that stresses the conceptualization of medical mistrust as occurring within inequitable systems and not within specific groups of people, such as self-identified race, ethnicity, or socioeconomic groups [64,65]. Future research should broaden its scope to look at other populations of patients with high and low medical mistrust to assess whether there are predictors beyond sociodemographic characteristics.

In alignment with similar research, a specific concern of our participants was the uncertainty surrounding the access and use of genetic data by external entities [45], with higher concerns associated with high medical mistrust. What remains unclear from this finding is whether TGP concerns related to use of genetic data and/or tumor genes originate from specific concerns related to self or immediate family members, or more general concerns about genetics and genes. It is also not known if these TGP concerns about tumor genetic data arise from less knowledge and/or genetic literacy in differentiating tumor genes from hereditary/germline DNA. Given that limited health literacy, cancer health literacy, and genetic literacy are especially prevalent among underserved and minority populations, including Black and AA cancer patients [32,33], these combined limited literacies warrant special attention within clinical settings. Additionally, their connection to higher levels of medical mistrust is an important research priority as access to genetic testing improves among underserved individuals.

The remaining three categories of concern that differed by medical mistrust cluster were the clinical utility and accuracy of genetic test results, the quality of providers’ communication about inconclusive test results, and the physical discomfort (i.e., pain) associated with the genetic testing procedure itself. Fears of pain associated with medical procedures, including needle pain, have been previously linked to the more general notion of distrust of healthcare providers, as repeated negative experiences with pain and physical discomfort in healthcare settings becomes associated with the healthcare setting and related providers [66]. Underlying factors that connect individuals with greater mistrust to beliefs that TGP has less clinical utility (e.g., TGP results might not help treat cancer) should also be further explored in psychometric studies. For example, concerns about TGP could be explored as a multidimensional construct that discriminates between beliefs in the effectiveness of TGP from mistrust of TGP, including genetic data being used for secondary purposes.

While there were small but statistically significant differences in oncologists’ communication skills between clusters, both groups maintained average ratings of provider communication skills as either neutral or positive (i.e., 6 or lower on the 1 to 11 scale). This is consistent with the related finding that there were no differences for two of the three indicators of patient–oncologist relationship quality by medical mistrust cluster. In general, study participants reported very good quality relationships with their oncologists (mean rating of 10.6 out of 11 in feeling comfortable talking with their oncologist; mean of 10.5 out of 11 in feeling heard and unrushed by their oncologist), suggesting that TGP concerns associated with medical mistrust may be more closely related to the trustworthiness of broader systems of healthcare, payors, and scientific institutions, rather than to the trustworthiness of their own oncologists and/or medical providers. In fact, past research has established that the quality of patient–provider relationships can moderate the relationship between greater medical mistrust and healthcare engagement [67]. This points to an important opening for communication about TGP with patients with higher overall mistrust in which providers can leverage the trust patients have in them to assuage TGP concerns. Future research may also consider investigating whether information about TGP from more trusted health information sources, such as Black and AA healthcare providers, community organizations (e.g., faith-based organizations), and family members [68], could mitigate the association between medical mistrust and TGP concerns. Finally, one potential strategy for mitigating some of the disparities in cancer outcomes includes improved communication strategies for relaying cancer-related and genetic testing-related information to cancer patients with high medical mistrust and potentially limited health literacy [38]. For example, researchers have developed tailored methods of communicating more effectively with patients with lower levels of health literacy about TGP [69], including specialized training programs for health professionals [70]. However, uncertainty remains about best practices for communicating about TGP that addresses the unique needs of these patients.

### Limitations

These results should be considered in the context of some limitations. First, patients were recruited for a research study from specialty medical clinics, which limits generalizability to community members who might use health services less, who avoid using healthcare altogether, or who would be less willing to participate in a research study or clinical trial. Both treatment facilities were also located in or adjacent to a large US city, meaning that these results may be less applicable to patients in rural areas or regions with fewer oncology specialty resources.

Study participants were also all insured (though many through the federal Medicare and Medicaid programs), and so these results may not reflect those of an uninsured population. Participants were also mostly middle-aged adults (mean age of 58.7 years), and thus may not generalize to younger or older adults with cancer. There may have been additional moderating factors, such as differences in cancer health literacy or treatment awareness, or exposure to TGP health information among patients with more advanced cancers. Finally, while there were no differences in sociodemographic (except age), clinical, and relationship with oncologist variables by medical mistrust cluster, a larger sample using other statistical methods—such as multivariable regression—would allow for statistical adjustment to control for these potential covariates.

## 5. Conclusions

High medical mistrust among Black and African American adult oncology patients from Philadelphia, PA without a known history of tumor genomic profiling was associated with greater concerns about tumor genomic profiling than low medical mistrust. Levels of medical mistrust were not significantly associated with sociodemographic factors, except for age. Moreover, medical mistrust levels were also not associated with oncology-related clinical factors or relationship quality between patients and their oncologists.

Communication about tumor testing and treatment options with Black and AA cancer patients who experience greater medical mistrust may need to better focus on addressing concerns about TGP. Healthcare system changes to cancer care provision, including increased representation of Black and AA oncologists and genetic counselors, may further improve their treatment experiences and related treatment decisions about TGP, as would targeted materials, such as decision aids, that could increase informed decision making.

## Figures and Tables

**Table 1:** Medical mistrust items by medical mistrust cluster (*N* = 112).

	High Mistrust	Low Mistrust	Total		
	*n* = 68	*n* = 44	*N* = 112		
Medical Mistrust Items	*M* (SD)	*M* (SD)	*M* (SD)	*t* (*df*)	*p*-Value
I think that doctors do not always give patients all of the information they need to know.	7.31 (3.09)	3.00 (2.98)	5.63 (3.70)	7.246 (108)	<0.001
I do not trust medical researchers.	4.75 (2.93)	2.18 (2.08)	3.74 (2.91)	5.417 (109)	<0.001 ^a^
I believe racial/ethnic minorities are discriminated against in medical research studies.	6.65 (2.77)	2.45 (2.41)	5.00 (3.33)	8.231 (110)	<0.001
I do not trust drug (pharmaceutical) companies.	8.15 (2.51)	3.45 (2.36)	6.30 (3.36)	9.891 (110)	<0.001
The government cannot be trusted to regulate use of genetic information.	8.44 (2.07)	4.77 (2.92)	7.00 (3.02)	7.785 (110)	<0.001 ^a^
Researchers do harmful experiments without a patient’s knowledge.	6.62 (2.55)	3.64 (2.71)	5.45 (2.98)	5.900 (110)	<0.001 ^a^
Medical research on minorities is just being done to make money.	5.58 (2.98)	3.07 (2.33)	4.59 (2.99)	4.662 (107)	<0.001
I think that medical researchers use minorities as guinea pigs.	6.03 (2.97)	3.16 (2.53)	4.90 (3.12)	5.223 (107)	<0.001
I do not trust insurance companies.	7.36 (2.72)	4.23 (3.21)	6.14 (3.29)	5.477 (108)	<0.001
I think that insurance companies prioritize profits over peoples’ health	9.16 (1.92)	5.77 (3.62)	7.84 (3.17)	6.408 (108)	<0.001 ^a^

Notes. *N* = total number of responses; *n* = number of responses per cluster. Response values ranged from 1 to 11 for each item. *M* = mean; SD = standard deviation; *t* = test statistic for independent samples t-test. ^a^ Levene’s test was significant, so equal variances were not assumed.

**Table 2:** Characteristics of study participants by medical mistrust cluster (*N* = 112).

		High Mistrust	Low Mistrust	Total		
		*n* = 68	*n* = 44	*N* = 112		
Characteristic		% (n)	% (n)	% (n)	χ^2^/t	*p*-Value
Sociodemographic Variables						
*Gender*	Male	23.5% (16)	18.2% (8)	22.2% (24)	0.45 (1)	0.501
	Female	77.8% (52)	81.8% (36)	77.8% (88)		
*Educational Attainment*	High school graduate or less	30.9% (21)	45.5% (20)	36.6% (41)	3.97 (2)	0.137
	Vocational school or some college	26.5% (18)	29.5% (13)	27.7% (31)		
	College graduate or more	42.6% (29)	25.0% (11)	35.7% (40)		
*Annual Household Income*	<USD 10,000	19.0% (11)	24.3% (9)	21.1% (20)	1.02 (3)	0.796
	USD 10,000–25,000	24.1% (14)	24.3% (9)	24.2% (23)		
	USD 25,001–75,000	32.8% (19)	35.1% (13)	33.7% (32)		
	>USD 75,000	24.1% (14)	16.2% (6)	21.1% (20)		
*Insurance*	Medicaid only	13.2% (9)	13.6% (6)	13.4% (15)	0.01 (2)	0.997
	Medicare or dual eligibility	32.4% (22)	31.8% (14)	32.1% (36)		
	Other insurance	54.4% (37)	54.5% (24)	54.5% (61)		
*Age, in years; M(SD)*		60.9 (9.8)	55.4 (10.2)	58.7 (10.3)	2.76 (109)	0.006
Clinical Factors						
*Oncology Treatment Facility*	Temple Hospital	33.8% (23)	27.9% (12)	31.5% (35)	0.61 (2)	0.788 ^a^
	Fox Chase Cancer Center	52.9% (36)	60.5% (26)	55.9% (62)		
	Both facilities	13.9% (9)	11.6% (4)	12.6% (14)		
*Cancer Stage*	Stage 1 through Stage 3	38.8% (26)	28.6% (12)	34.9% (38)	3.38 (3)	0.336
	Stage 4 (Advanced/Late Stage)	22.4% (15)	38.1% (16)	28.4% (31)		
	Cancer survivor	16.4% (11)	11.9% (5)	14.7% (16)		
	Unsure	22.4% (15)	21.4% (9)	22.0% (24)		
*Cancer History*	First cancer diagnosis	76.4% (55)	85.0% (34)	79.5% (89)	1.17 (1)	0.280
	Previously had cancer	23.6% (17)	15.0% (6)	20.5% (23)		
Relational Factors						
*Feels comfortable talking with own oncologist; M(SD)*		10.4 (1.6)	10.8 (0.5)	10.6 (1.3)	−2.27 (109)	0.026 ^b^
*Feels heard and unrushed by own oncologist; M(SD)*		10.4 (1.3)	10.6 (1.5)	10.5 (1.3)	−0.78 (110)	0.440
*Own oncologist listens to patients equally regardless of race; M(SD)*		9.5 (2.1)	10.1 (2.0)	9.8 (2.1)	−1.60 (108)	0.114 ^b^

Notes. *N* = total number of responses; *n* = number of responses per cluster. *M* = mean; *SD* = standard deviation; χ^2^ = chi-squared test of independence; *t* = independent samples t-test. Missing or invalid responses <5% were excluded. 17 missing/invalid responses for income were excluded; missing income did not differ between groups (14.7% (*n* = 10) for MM-H; 15.9% (*n* = 7) for MM-L). Dual eligibility = individuals had Medicare and Medicaid. ^a^ Fisher’s exact test was used for significance testing because one cell or more had an expected count of fewer than five. ^b^ Levene’s test was significant, so equal variances were not assumed.

**Table 3:** Concerns about tumor genomic profiling by high versus low medical mistrust clusters (*N* = 112).

	High Mistrust	Low Mistrust	Total		
	*n* = 68	*n* = 44	*N* = 112		
Concerns about TGP	*M* (SD)	*M* (SD)	*M* (SD)	*t (df)*	*p*-Value
Concerned about what my tumor genetic test might show.	6.85 (3.25)	6.07 (3.85)	6.54 (3.50)	1.160 (110)	0.249
Tumor genetic test might be costly.	7.75 (2.91)	5.75 (3.58)	6.96 (3.32)	3.241 (110)	0.002
Tumor genetic test might result in insurance discrimination.	7.31 (3.00)	4.73 (3.27)	6.29 (3.34)	4.296 (110)	<0.001
My genetic information might be shared with others if I have a tumor genetic test.	5.94 (2.99)	4.05 (3.09)	5.19 (3.16)	3.224 (109)	0.002
My tumor genetic test results might not help treat my cancer.	6.13 (3.22)	3.73 (3.06)	5.21 (3.35)	3.861 (110)	<0.001
My tumor genetic test might not guarantee that a new medication will work for my cancer.	6.93 (3.24)	4.64 (3.01)	6.02 (3.33)	3.750 (109)	<0.001
My tumor genetic test might not provide accurate information.	6.35 (2.82)	4.48 (2.94)	5.62 (3.00)	3.381 (110)	0.001
My doctor might not be able to explain the results of my tumor genetic test if it has uncertain results.	5.79 (3.14)	4.43 (3.25)	5.26 (3.24)	2.213 (110)	0.029
Results of my tumor genetic test might be used by researchers without my knowledge.	5.84 (3.48)	3.12 (2.43)	4.77 (3.38)	4.817 (107)	<0.001 ^a^
Results of my tumor genetic test might mean my family is at risk of getting cancer.	5.39 (3.53)	4.17 (3.30)	4.93 (3.48)	1.784 (106)	0.077
Tumor genetic test would be painful.	5.72 (3.11)	3.93 (3.06)	5.05 (3.20)	2.902 (105)	0.005

Notes. Number of valid/non-missing responses for each item ranged from 107 to 112. Differences in means between clusters were tested using independent samples *t*-tests. *N* = total number of responses; *n* = number of responses per cluster; *M* = mean; SD = standard deviation; *t* = independent samples *t*-test; *df* = degrees of freedom. ^a^ Levene’s test was significant, so equal variances were not assumed.

## Data Availability

Data available from the corresponding author upon reasonable request.
